# Harnessing Clinical Trial and Real-World Data Towards an Understanding of Sex Effects on Drug Pharmacokinetics, Pharmacodynamics and Efficacy

**DOI:** 10.3389/fphar.2022.874606

**Published:** 2022-06-06

**Authors:** Joyce Oi Yan Chan, Marie Moullet, Beth Williamson, Rosalinda H. Arends, Venkatesh Pilla Reddy

**Affiliations:** ^1^ Clinical Pharmacology and Safety Sciences, Biopharmaceuticals R&D, AstraZeneca, Cambridge, United Kingdom; ^2^ UCB UK Ltd, Slough, United Kingdom; ^3^ Clinical Pharmacology and Safety Sciences, Biopharmaceuticals R&D, AstraZeneca, Gaithersburg, MD, United States

**Keywords:** clinical pharmacology, drug metabolism, pk, sex, POPPK

## Abstract

Increasing clinical data on sex-related differences in drug efficacy and toxicity has highlighted the importance of understanding the impact of sex on drug pharmacokinetics and pharmacodynamics. Intrinsic differences between males and females, such as different CYP enzyme activity, drug transporter expression or levels of sex hormones can all contribute to different responses to medications. However, most studies do not include sex-specific investigations, leading to lack of sex-disaggregated pharmacokinetic and pharmacodynamic data. Based available literature, the potential influence of sex on exposure-response relationship has not been fully explored for many drugs used in clinical practice, though population-based pharmacokinetic/pharmacodynamic modelling is well-placed to explore this effect. The aim of this review is to highlight existing knowledge gaps regarding the effect of sex on clinical outcomes, thereby proposing future research direction for the drugs with significant sex differences. Based on evaluated drugs encompassing all therapeutic areas, 25 drugs demonstrated a clinically meaningful sex differences in drug exposure (characterised by ≥ 50% change in drug exposure) and this altered PK was correlated with differential response.

## Introduction

Personalised medicine aims to improve drug efficacy and minimize side effects by tailoring treatment to specific populations. While sex is a basic biological factor driving differences between individuals (Wagner et al., 2019), women are often under-represented in clinical studies. Reasons include potential interactions between new treatment, hormonal fluctuations and oral contraceptives (Chu, 2014). Accumulating evidence supports that physiological differences between males and females, such as different body composition, metabolic enzyme and transporter activities and other sex-related disparities can contribute to different responses during drug treatment (Gandhi et al., 2004; Soldin and Mattison, 2009; Chu, 2014).

Sex differences in metabolic enzyme activity are one potential mechanism for sex differences in pharmacokinetics (PK). A previous review of the influence of age and sex on CYP3A substrates found that there were no significant differences in clearance of the majority of CYP3A4 substrates between males and females (Cotreau et al., 2005). Where there was a statistically significant difference, females tended to have higher clearance than males. There was heterogeneity in reported effects even when comparing studies of the same compound, for example verapamil had higher clearance in females in three out of five identified studies. Variation between studies could be confounded by differences in other variables such as route of administration. CYP3A metabolism may additionally depend on interactions with p-glycoprotein transporter (Cummins et al., 2003). Heterogeneity in study design and biological interactions pose a challenge for the identification of general principles [underpinning sex differences] emphasize the need for more detailed studies of physiological mechanisms driving sex differences.

A recent study demonstrated that sex-biased pharmacokinetics predict Adverse Reactions (ADR) in women for certain drugs (Zucker and Prendergast, 2020). Prescribing the same dose to men and women might be appropriate considering the physiological differences and resultant clinical effects (Gandhi et al., 2004). Hence, sex-based dose adjustment is required to minimise the risk of developing ADR in women (Zucker and Prendergast, 2020). An example of drug that applies sex-based dose adjustment is zolpidem. For a given dose of zolpidem, women are at a greater risk of adverse drug reactions (ADR) due to the lower metabolic clearance (CL) of zolpidem (Chu, 2014; Zucker and Prendergast, 2020).

AstraZeneca’s “5R” framework aims to accelerate drug development by identifying the right target, the right patient, the right tissue, the right safety and the right commercial potential early in the drug discovery process (Cook et al., 2014). Taking sex differences in PK and Pharmacodynamics (PD) into account can ensure drugs are given to the right patient and the right population with the right safety. However, despite an increased enrolment of women, most clinical studies do not include sex-specific analyses (Chu, 2014; Zucker and Prendergast, 2020). For many compounds, including anticancer drugs, sex differences in the dose concentration-drug response relationship and the mechanisms underlying the sex differences are largely unknown (Zucker and Prendergast, 2020).

A multidisciplinary workshop conducted by the European Society for Medicine Oncology highlighted the impact of sex on the PK of chemotherapeutic agents and underlined the need to conduct a systematic literature review on PK sex-related differences of all types of anticancer drugs (Wagner et al., 2019). Hence, the aims of the present literature review were three-fold: to highlight drugs with significant sex PK differences, to investigate the clinical impact of the sex differences with respect to PD, efficacy and ADR, and to explore potential mechanistic explanations for sex differences in order to guide future research directions. To achieve this, we identified anticancer and non-anticancer drugs with ≥50% sex-differences in drug exposure and studied how this PK difference propagated to PD, efficacy and ADR differences.

## Materials and Methods

### Sex-Stratified PK Data Collection

Firstly, we reviewed the sex differences in PK of anticancer drugs. Multiple searches in NCBI PubMed were conducted using the following search terms: *oncology drugs, anticancer drugs, sex differences in pharmacokinetics* or *gender differences in pharmacokinetics*. In addition to the keyword search, a list of marketed anticancer drugs were obtained from the Washington database (90 anticancer drugs in total). Individual searches for each anticancer drug were then run in PubMed with the following search terms: *“name of the drug” and sex differences and pharmacokinetics, “name of the drug” and gender differences and pharmacokinetics.*

We then expanded our search to non-cancer agents by searching for the following terms: s*ex differences in pharmacokinetics* and *gender differences in pharmacokinetics.* Only English language publications that reported sex-stratified PK data and their variance were included and no date restrictions were applied to search results.

Sex-stratified PK data were manually extracted from relevant publications, weight-normalised PK data were used if available. If results were only presented graphically or not reported in University of Washington Drug Interaction Database, concentration time profiles were digitized using AstraZeneca internal graph reader tool (Version 4.1) in order to estimate PK parameters using a compartmental approach. Trial design and patient demographics were recorded. Drugs were considered to have a significant difference in PK if the female to male ratio for a given PK parameter was greater than 50% and the *p*-value was smaller than 0.05, after adjusting for body weight. When the 90% confidence interval (CI) of the sex ratio estimate fell within the bioequivalence window (0.80–1.25), we considered that the sex effect may not be clinically significant.

### Literature Search and Sex-Stratified PD Collection

Clinical outcomes (changes in PD, efficacy and ADR) associated with the sex-biased PK identified in the previous section were then investigated. The impact of sex on PD effects were only studied for drugs with ≥50% sex differences in exposure identified previously that do not produce active metabolites. The following key words were searched in PubMed with no year restrictions: *sex differences* or *gender differences in “pharmacodynamics”*,“*efficacy”*, “toxicity”, *“safety”, “adverse drug reactions” or “side effects”.* Only English language publications that contain sex-stratified PD, efficacy or toxicity data were retained. Additional sex differences in ADR incidence were searched using Vigibase (https://who-umc.org/vigibase/), applying similar methods to those described in Zucker et al., 2020 (Zucker and Prendergast, 2020). Briefly, we searched Vigibase for each of the drugs for which we identified significant sex-differences in PK. We then recorded the female to male ADR ratio reported in Vigibase and examined the concordance with the direction of sex-biased PK reported in the previous section. We considered a female to male ratio above 1.25 or below 0.8 as a clinically significant sex difference in ADR incidence.

## Results

### Sex Disparities in PK of Anticancer Drugs

Among the searched anticancer drugs, the sex differences in PK were significant for 13 drugs (Supplementary Figure S1). These compounds included six biologics and four kinase inhibitors. The remaining compounds were antineoplastic hormonal drugs including, anthracyclines and antimetabolites therapeutics (Figure 2 and Supplementary Table S1). The sex differences of these anticancer drugs are characterised by lower CL and volume of distribution (Vd) in females. The 90% CI of the reported PK parameters for most of these anticancer drugs, except for tamoxifen, doxorubicin and rituximab, included the bioequivalence window, suggesting their sex differences in CL and Vd may not be clinically significant. However, only tamoxifen and doxorubicin had a percentage change ≥50% (108 and 60%, respectively) (Figure 2).

**FIGURE 1:**
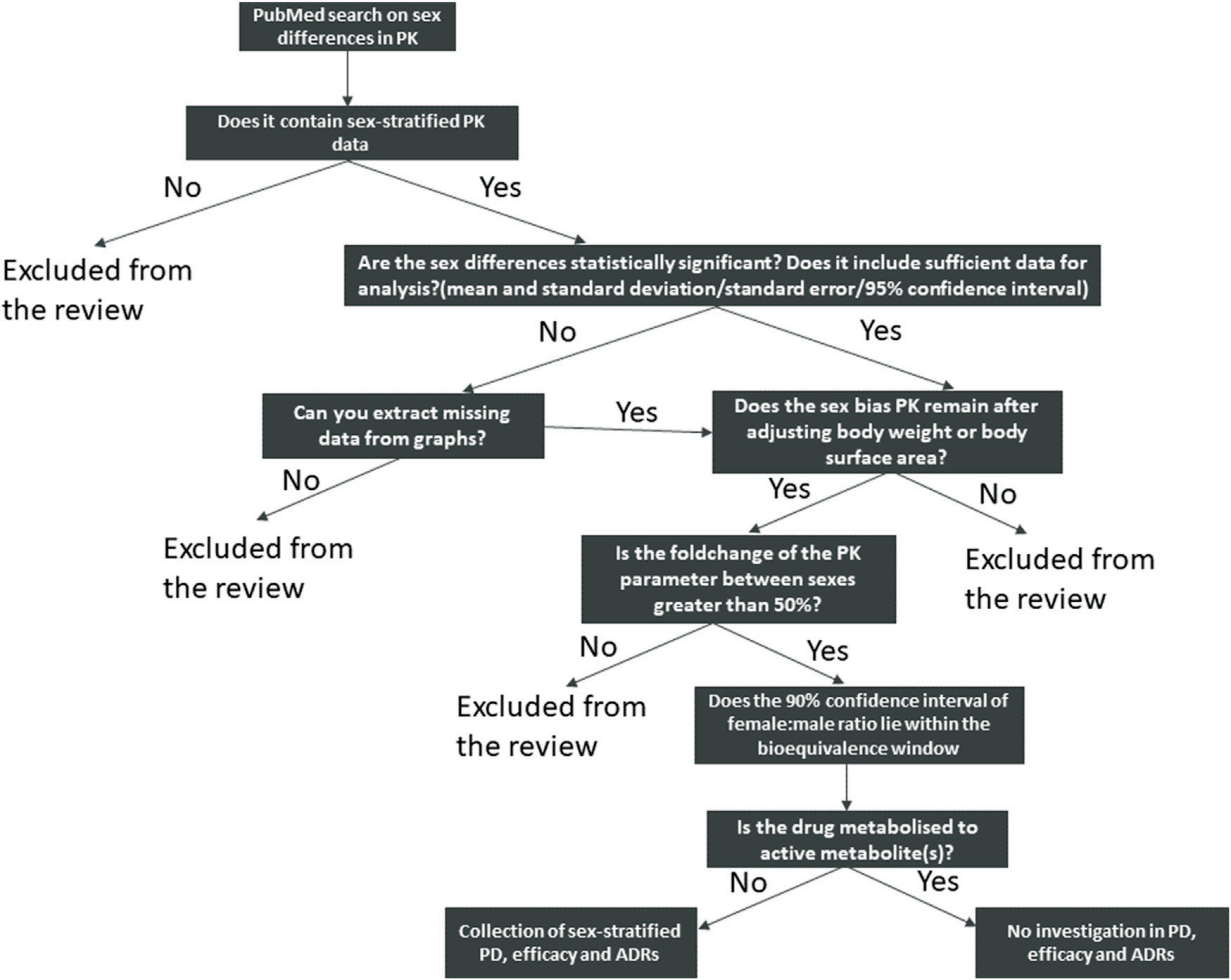
Schematic view of the search strategy and identification of the drugs included in this review.

**FIGURE 2:**
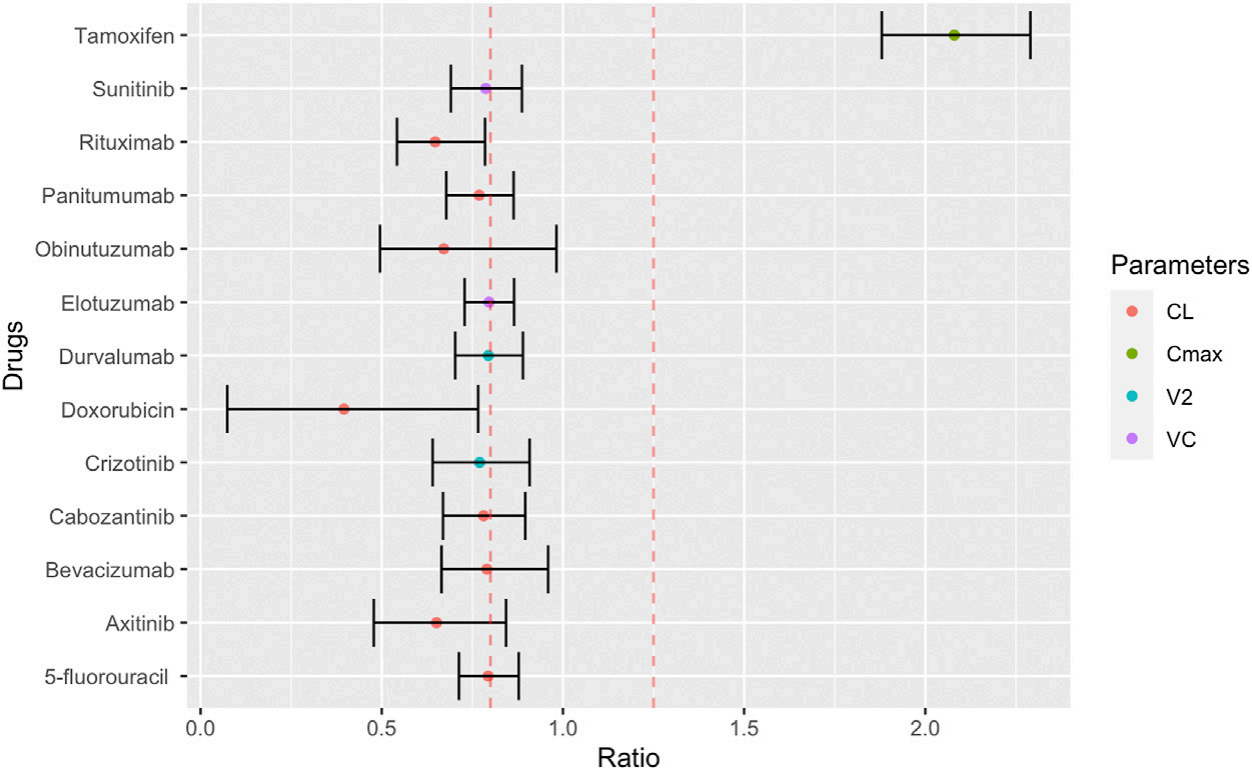
Female to male ratio of observed PK parameters of anticancer drugs. The ratio ranges from 0.40 (smaller PK value in women) to 2.29 (larger PK value in women) with the 90% confidence interval indicated by error bars. Red dashed lines indicate bioequivalence window from 0.8 to 1.25. PK abbreviations: clearance (CL), maximum plasma concentration (Cmax), volume of distribution to peripheral compartments (V2), volume of distribution of central compartment (VC).

### Sex Disparities in PK of Drugs From Other Therapeutic Areas

**FIGURE 3:**
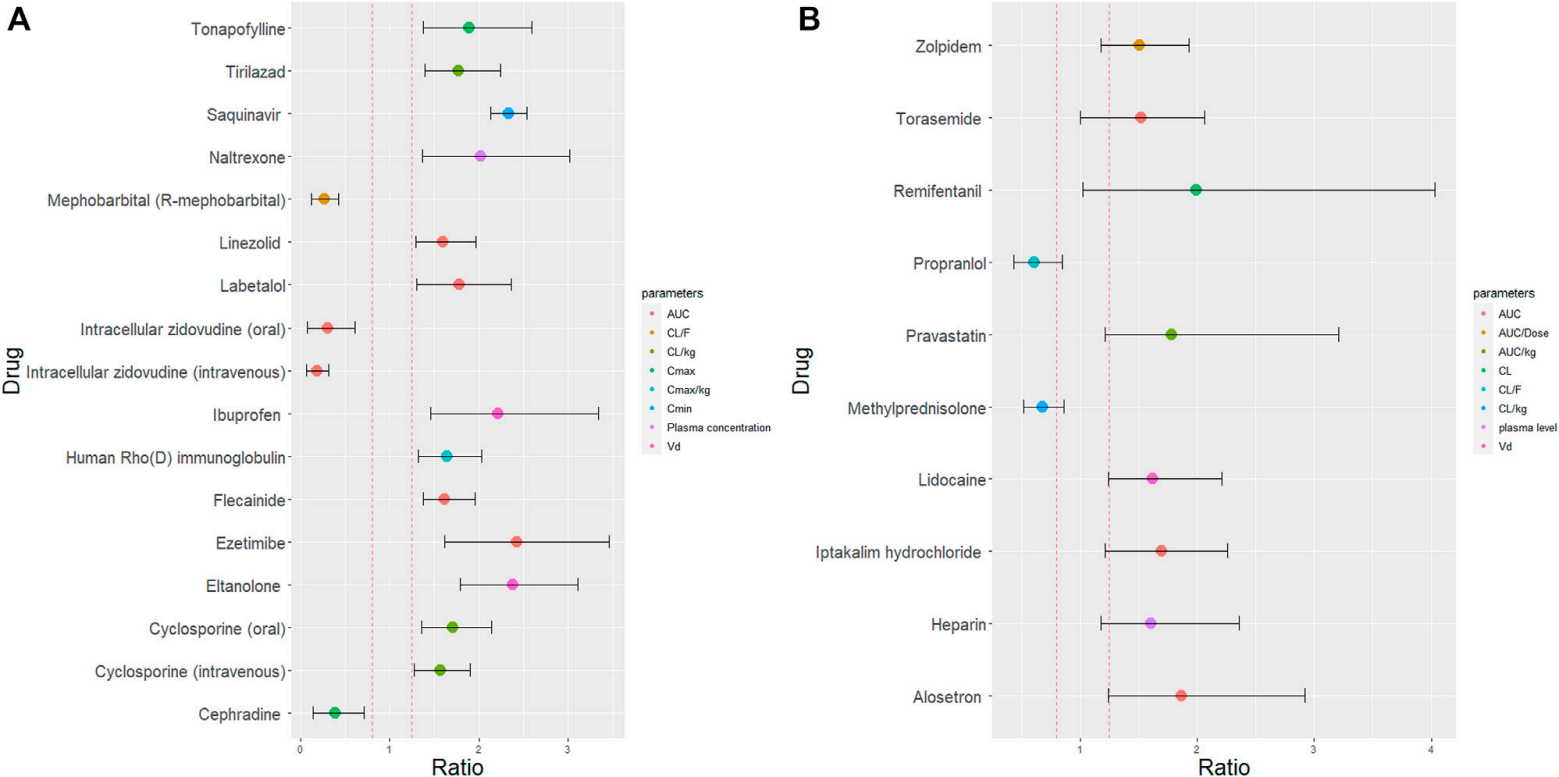
Female to male ratio of observed PK parameters of drugs with at least 50% sex differences in PK. The ratio ranges for all 25 drugs from 0.19 (smaller PK value in women) to 2.38 (higher PK value in women). Red dashed lines indicate the bioequivalence window from 0.8 to 1.25. The 90% confidence interval are indicated by the error bars which either fell outside the bioequivalence window **(A)** or inside the bioequivalence window **(B)**. Where, AUC is area under curve; CL is clearance (corrected for body weight) (CL/kg); CL/F is oral clearance; Cmax is maximum plasma concentration (corrected for body weight) (Cmax/kg); Cmin is trough plasma concentration and Vd is volume of distribution.

We identified 25 drugs with greater than 50% change in PK (maximum concentration (Cmax), area under the curve (AUC) and CL, Figure 3). Patient demographics and study design are summarised in Supplementary Table S2 and Supplementary Table S3). Among these 25 drugs, 24 were small molecules and 1 was a biological drug (human Rho(D) immunoglobulin). Of these drugs, 3 (tonapofylline, mephobarbital (R-mephobarbital) and elthanolone) do not currently have FDA approval. For 21/25 drugs, females exhibited higher Cmax and AUC than males. The 90% CI of the female to male ratio excluded the bioequivalence window for 15 out of 25 drugs suggesting these differences could be clinically significant (Figure 3A). The reported sex difference may not be clinically significant for the remaining 10 drugs (Figure 3B).

### Impact of Sex-Related Differences in PK on PD, Efficacy and ADR During Cancer Treatment

In the previous section, we identified drugs with previously reported sex differences in multiple PK parameters. However, the impact on drug efficacy and safety will ultimately define the need or requirement for dose adjustments.

We identified two cancer drugs with a sex difference of ≥50% for at least one PK parameter. The efficacy and safety of doxorubicin and tamoxifen depends on the metabolism of their active metabolites, doxorubicinol (Olson et al., 1988)and endoxifen (Jager et al., 2014).

### Impact of Sex-Related Differences in PK on PD, Efficacy and ADR in Other Therapeutic Areas

Drugs identified from non-oncology therapeutic areas with a reported sex difference of ≥50% for at least one PK parameter of the parent drug. Active metabolite data is not considered in this review due to limited reported information. The clinical impact of the sex differences could not be established for eltanolone as PD studies were completed in rat only. To our knowledge, no literature exists reporting the impact of sex on the PD of human rho(D) immunoglobulin and iptakalim hydrochloride. These compounds were also were absent from Vigibase. Of the remaining drugs, the direction of the sex differences in PK and PD (female exposure > male exposure) were the same for six drugs (Table 1). Eight drugs had ADR data available in Vigibase, 4 of them had significant sex differences in the same direction. The sex-specific effects in PD, efficacy and ADR of individual drugs where available are discussed in more detail below.

**TABLE 1:** Summary of sex biased-PK, sex-biased PD, sex report ratio from Vigibase and concordance of PK with PD and clinical ADR.

Drug	Therapeutic Class	PK parameter^a^	PD parameter^b^	Vigibase Report Ratio	Concordance^c^ (PK vs. PD)	Concordance^c^ (PK vs. vigibase)
Alosetron	5-HT3 receptor antagonist	**AUC**	**Percentage responders** Pain free days Brain serotonin (5-HT) synthesis	11.24	Concordant	Concordant
Cephradine	Antibiotics	Cmax	N/A	1.20	N/A	NS
Eltanolone	Anaesthetics	**Vd**	**Induction time** Sleeping time **LD50**	N/A	Concordant	N/A
Linezolid	Antibiotics	**AUC**	Number of patients with linezolid-associated thrombocytopenia	0.74	Discordant	Discordant
Methyl prednisolone	Immunosuppressive	**Weight-adjusted CL**	IC50 for cortisol suppression Area between the base line and effect curves for basophil trafficking Zero-order of return of basophils from the extravascular compartment	1.35	Concordant	Concordant
Pravastatin	Statin	**Weight-adjusted AUC**	Changes in thrombogenicity markers^1^ Changes in lipid values^1^	1.12	Discordant	NS
Saquinavir	Antiviral	**Cmin**	**Probability of having significant effect on week 16 HIV RNA ≤500 copies Inhibitory quotient (AUC/IC50)**	2.52	Concordant	Concordant
Unfractionated heparin	Anticoagulant	**Plasma concentrations**	**APTT values Heparin-induced thrombocytopenia**	0.89	Concordant	NS
Zolpidem	Hypnotic	**AUC**	Digital Substitution Test (DSST) score **Number of lapses in Choice Reaction Test (CRT)** Symbol Copying Test (SCT) Sleep efficiency^1^ Wake after sleep onset^1^ Latency to persistent sleep^1^	1.46	Concordant	Concordant

Where, AUC is area under the curve; Cmax is maximum plasma concentration; Vd is volume of distribution; CL is clearance; N/A highlights absent data and NS is not significant.

a Bold font indicates higher value of the corresponding PK parameter in females than males

b Bold font indicates higher value of the corresponding PD parameter in females than males

c It is sex-concordant when the direction of PK drug exposure is the same as the direction of PD effect/sex report ratio from Vigibase.

1 No differences in the corresponding PD parameter between females and males.

### Discussion of Potential Mechanisms Biological Mechanisms

The molecular mechanisms underpinning sex differences in PK are likely to be drug-specific. In the following section, we discuss hypotheses for the biological mechanisms which may be contributing to the sex difference in PK. As noted, we focused on drugs (Figure 2 and Figure 3) which demonstrated ≥50% sex difference in at least one PK parameter. Where sex-specific PD data is available, we explore the concordance between sex-specific changes in PK and PD and its link to ADR or efficacy. However, disease-associated inflammation can induce changes in CYP and transporter expression levels and could be at a different levels in females compared to males. (Schwenger et al., 2018). There are no references in the literature to support the disease-altered drug metabolism may also depend on sex differences in disease that in turn, alter the PK of certain compounds during the disease condition.

### Cancer Drugs

Among the cancer drugs highlighted in Figure 2, doxorubicin and tamoxifen demonstrate a sex-specific effect of ≥50% for at least one PK parameter. We therefore concentrated on the biology of these two drugs.

Doxorubicin is a commonly prescribed cytotoxic drug for solid tumours (Tacar et al., 2013; He et al., 2018), but exhibits cardiotoxicity in the clinic which is associated with the accumulation of its active metabolite, doxoubicinol (Olson et al., 1988; Tacar et al., 2013). Doxorubicin is mainly metabolised by the cytochrome P450 enzyme (CYP) 2B1, and its CL predominantly depends on biliary elimination via two major efflux transporters, P-glycoprotein (P-gp) and multidrug resistance protein 2 (MRP2) (Suzuki et al., 2006). Dobb et al., 1995, found that female subjects demonstrated lower total doxorubicin CL (defined as dose/AUC), while male subjects demonstrated higher CL resulting in lower concentration of the parent drug but higher concentrations of active metabolite, doxoubicinol. The authors did not examine sex differences in hepatobiliary and renal CL (Dobbs et al., 1995). In humans, hepatic P-gp expression is reported to be higher in males (Schuetz et al., 1995). Taken together, these studies suggest doxorubicin may be mainly effluxed by P-gp in males which could contribute to the increased CL reported by Dobb et al., 1995. Differences in transporter expression between sexes have previously been linked with different levels of sex hormone (Schuetz et al., 1995; Suzuki et al., 2006). From these data we can hypothesize that hormone-driven differences in drug transporter expression contribute to sex-differences in PK. Renal CL also accounts for 12% of doxorubicin total CL (Pippa et al., 2020). As such, the combination of sex-specific expression of drug transporters in both liver and kidney with doxorubicin PK from the same study in humans would help to elucidate the biological mechanisms of doxorubicin sex differences.

Tamoxifen is an adjuvant therapy and chemotherapy for oestrogen receptor-positive breast cancer (Jager et al., 2014). Male breast cancer is a rare condition, leading to lack of prospective trials and data (Eggemann et al., 2020). Sex differences in tamoxifen PK were reported in the FDA regulatory submission, which compared PK parameters in healthy male and female subjects from two different clinical studies (Food and Drug Administration, 2005). Maximum concentration (Cmax) was higher in females than males. However, comparing these clinical studies may not be appropriate due to variation in study design or patient demographic characteristics (age range, ethnicity and use of concomitant medications). Sex differences in the PK of tamoxifen and its metabolite have not been specifically studied. This example illustrates the importance of appropriate study design to allow exploration of sex differences in cancer drug metabolism.

### Other Therapeutic Areas

In the following section, we discuss the non-cancer drugs for which we identified ≥50% sex difference in at least one PK parameter. Where PD and ADR data was available, we compare the direction of sex-effects in PK and PD/ADR. These case studies suggest sex differences in metabolic enzyme and transporter activity or body composition may play a role in driving sex differences in PK, potentially interacting with genotype or other patient demographic characteristics such age and ethnicity. We also highlight examples where insufficient data is available, hindering the ability to understand or generate hypotheses of the mechanism of action or two link differences in PK to effect on efficacy and safety.

Saquinavir is an antiretroviral drug with very poor oral bioavailability due to limited intestinal absorption and high first pass metabolism (Vella and Floridia, 1998; Fletcher et al., 2004). Fletcher et al., 2004, showed that the AUC and Cmin of saquinavir were significantly higher in female than male subjects (Fletcher et al., 2004). This is consistent with another Phase I study in which females exhibited significantly higher saquinavir AUC and Cmax than males (Pai et al., 2004). Hepatic CYP3A4 accounts for 90% of saquinavir metabolism, and CYP3A4-intestinal metabolism also contributes to the low oral bioavailability of saquinavir (Vella and Floridia, 1998; Mouly et al., 2004; Lledó-García et al., 2011). While sex differences in CYP3A4 activity could contribute to observed PK differences, clinical studies have focused on investigating the co-administration of saquinavir with two irreversible CYP3A4 inhibitors (Fletcher et al., 2004). Therefore any differences in CYP3A4 activity cannot be fully elucidated and the mechanistic reasons for the sex differences remain unknown. Increased male expression of the drug efflux pump, P-gp, in the intestine (Mai et al., 2021) could also contribute to lower AUC and Cmin in males. In addition, sex differences could also be due to differences in plasma protein levels: for example, alpha-1 acid glycoprotein (AAG) binds saquinavir in plasma (Vella and Floridia, 1998; Fletcher et al., 2004) and decreases in response to estrogen (Gandhi et al., 2004; Mouly et al., 2004; Soldin and Mattison, 2009). Differences in AAG levels and binding could lead to different distribution of saquinavir between sexes. Thus, sex differences in saquinavir PK could be the product of hormonal effects on drug transporters and plasma glycoprotein levels.

In Fletcher et al., 2004, increased saquinavir exposure in females correlated with improved virologic response (Fletcher et al., 2004). Females had a greater likelihood of achieving HIV RNA level ≤500 copies compared to males. The inhibitory quotient (IQ) levels were also higher in females indicating enhanced virologic response (Vella and Floridia, 1998; Fletcher et al., 2004). Though ADR associated with the use of saquinavir are usually mild and infrequent (Vella and Floridia, 1998), the sex ratio from Vigibase highlighted that females are more susceptible to saquinavir-induced ADR than males. This may be due to higher exposure to saquinavir (Fletcher et al., 2004). As such, saquinavir provides an example of concordance between direction of drug transporter levels, PK fold changes, efficacy measures and ADR. Measuring these outcomes in the same study could help support a causal link between these observations.

Alosetron is a selective serotonin 5-hydroxytryptamine (5-HT) receptor 3 antagonist (Koch et al., 2002), currently approved for females with diarrhoea predominant irritable bowel syndrome (IBS) (41). This sex-specific medication is due to a lack of efficacy observed in males (Koch et al., 2002). 5-HT is involved in the brain-gut interaction which plays an important role of pathophysiology of IBS (Nakai et al., 2005). In Koch et al., 2002, the AUC and Cmax were significantly higher in females than males after oral dosing (Koch et al., 2002). The first pass metabolic CL of alosetron may play a major role in driving the observed sex differences. The serum concentrations in female subjects after oral dosing were approximately 2-fold higher than male subjects. (Koch et al., 2002). However, the exposure sex differences were age dependent and observed in the elderly but not young adults (Koch et al., 2002). Differential metabolism of some CYP3A4 and CYP2C9 substrates has also been reported for the elderly in previous studies (Koch et al., 2002). Thus, the sex differences in serum concentration of alosetron in the elderly might be a consequence of different CYP activities, providing a second example of the interaction between age and sex on CYP activity.

Alosetron efficacy is significantly influenced by sex. Female IBS patients demonstrated substantial improvement of IBS symptoms (pain and bowel function, including stool consistency and number of days with urgency) during alosetron treatment compared to placebo (Mangel and Northcutt, 1999) however no improvements were shown in male patients at any dose of alosetron (Mangel and Northcutt, 1999). However, the lack of efficacy in males could be due to an under-representation of men in clinical studies or true biological effect linked to increased 5-HT synthesis in males (Nakai et al., 2005). These studies did not investigate the interaction between age and sex on PD, and it is not known whether sex differences in alosetron plasma concentration are related to differences in brain 5-HT synthesis. The Vigibase sex ratio supports 11.2 times higher ADR incidence in women, though this result is biased as alosetron is only prescribed to women.

Zolpidem is a hypnotic drug used to treat insomnia and is one of the few drugs on the market that requires a dose adjustment based on sex; 50% reduction of maximum dosage approved for men is administered to women (Greenblatt et al., 2014). In Greenblatt et al., 2014, zolpidem exposure was significantly higher in females after sublingual dosing (Greenblatt et al., 2014). However, there is a lack of data to understand the mechanism underlying the sex differences in sublingual absorption. Previous studies have reported that the drug is deposited to the mucosal membrane, which leads to different systemic exposure after sublingual dosing (Bartlett and van der Voort Maarschalk, 2012). We hypothesize that there might be sex differences in drug partition to the mucosal membrane, resulting in different systemic exposure of zolpidem between sexes. In Greenblatt et al., 2014, the efficacy of zolpidem was significantly higher in women across four cognitive tests (Greenblatt et al., 2014). Approximately 84% of zolpidem is bound to albumin (Ishibashi et al., 1993). Previous reports have noted lower levels of albumin in females which may result in higher unbound zolpidem in females (Denko and Gabriel, 1981). This could partly explain the observed sex differences in PD effects. Vigibase data also supports the higher ADR incidence recorded in females. However, other studies did not identify a statistically significant correlation between sex and efficacy (sleep efficiency, wake after sleep onset or latency to persistent sleep) or ADR incidence (Roehrs and Roth, 2016). These contrasting clinically statistically significant sex observations for a drug with a well-established sex-specific dose adjustment illustrates the importance of conducting well-powered studies in order to characterize the effect of sex on PK and PD.

Methylprednisolone is a synthetic gluco-corticosteroid used as an anti-inflammatory medicine due to its immunosuppressive effects (Lew et al., 1993; Sloka and Stefanelli, 2005). A PK study conducted by Lew et al., 1993, found that females exhibited significantly higher body weight-normalized CL than males (Lew et al., 1993). CYP3A isoforms are involved in the metabolism of methylprednisolone, and previous studies have showed higher levels and activity of hepatic CYP3A4 in females. While the effect of sex on clearance on CYP3A substrates varies across compounds and studies (Cotreau et al., 2005), sex differences in CYP enzymes may contribute to greater clearance of methylprednisolone (Gandhi et al., 2004; Zucker and Prendergast, 2020). Methylprednisolone is a substrate of P-gp, males have higher hepatic P-gp levels than females (Gandhi et al., 2004), this might result more methylprednisolone being exposed to the CYP3A enzymes in females, and explain greater clearance observed in females.

Lew et al., 1993, also explored sex differences in methylprednisolone-induced immune suppression by examining the effect on cortisol, histamine and helper T lymphocytes (Lew et al., 1993). The study team observed a significantly lower 50% inhibitory concentration (IC_50_) for cortisol suppression, and lower IC_50_ for histamine (a marker of basophil suppression) and helper T lymphocytes (though the difference was not statistically significant). The duration of cortisol suppression in females was similar to males, likely a consequence of increased elimination rate in females (Lew et al., 1993). As a result, the net cortisol suppression was similar in both sexes. Previously studies had reported higher basal corticotropin concentration in men (a hormone which stimulates cortisol release) (Horrocks et al., 1990). This suggested that the female adrenal cortex might be more sensitive to corticotropin. Thus, methylprednisolone could produce greater cortisol suppression effect in females with equivalent reduction of corticotropin as males. (Kong et al., 1989; Lew et al., 1993). However, Lew et al., 1993, did not identify sex differences in corticotropin plasma levels. Thus the mechanism underlying sex differences in suppressive effect on cortisol of methylprednisolone has not been fully elucidated.

The majority of circulating histamine (at least 98%) can be found within the basophils (Wald et al., 1991), therefore, the reduction of histamine can be correlated to decrease in basophils. Methylprednisolone suppressive effect on basophils was negatively correlated with estradiol concentration in women, suggesting sex hormones may affect methylprednisolone efficacy. The sex report ratio in Vigibase is greater than 1.25, suggesting a female is more likely to experience methylprednisolone-induced side effects.

Pravastatin is a statin which is used to treat hypercholesterolemia (Niemi et al., 2006). It inhibits the 3-hydroxy-3-methylglutaryl-coenzyme A reductase in the hepatocytes to reduce cholesterol level (Niemi et al., 2006). The organic anion transporting polypeptide (OATP) 1B1 on the basolateral membrane of hepatocytes which is encoded by the *SLCO1B1* gene, aid the influx of pravastatin into hepatocyte (Niemi et al., 2006). Genetic variation in *SLCO1B1* is associated with changes in drug transporter activity and drug PK. In Niemi et al. (2006), AUC differences by sex were observed in subjects with the homozygous *SLCO1B1* c.521 TT genotype whereas there were no sex differences in other variants (c.521CC and c.521 TC) (Niemi et al., 2006). The sex differences in PK for individuals with c.521 TT may be due to resulting changes in intragastric acidity, which is higher in males than in females (Niemi et al., 2006; Soldin and Mattison, 2009). Higher intragastric acidity may increase acid-catalysed isomerisation and thus reduce plasma concentration of unchanged pravastatin in male subjects (Niemi et al., 2006). Pravastatin active efflux out of the liver and intestine is dependent upon multi-drug resistance protein (MRP) 2 as well as OATP1B1 (Niemi et al., 2006). However, there is currently insufficient data to suggest the mediation of sex differences in pravastatin PK is driven by MRP2. Dangas et al., 1999, did not identify sex differences in the efficacy of pravastatin (based on thrombus formation markers and lipid profiles) (Dangas et al., 1999). However, this could be because of the *SLCO1B1* polymorphisms which masked the sex effects. The ADR report sex ratio from the Vigibase fell within the bioequivalence window, suggesting the ADR incidences are similar in both sexes. These data suggest that the influence of sex on pravastatin PK and PD may be dependent on the patients *SLCO1B1* genotype, with sex differences observed only for one specific variant.

Cephradine is a beta-lactam antibiotic used to treat upper respiratory tract infections. Cephradine does not undergo hepatic metabolism and is excreted uncharged in the urine (Singhvi et al., 1978; Holten and Onusko, 2000). Vukovich et al., 1975, reported lower cephradine Cmax and AUC in females than males following intramuscular dosing to the gluteus maximus (Vukovich et al., 1975). Cephradine absorption may be greater in males due to greater muscular mass and organ blood flow (Gandhi et al., 2004; Soldin and Mattison, 2009; Schorr et al., 2018). Interestingly, no sex differences in PK were observed when the drug was administered to other sites, e.g. deltoid and vastus lateralis (Vukovich et al., 1975). This was an interesting observation as previous studies have shown that the deltoid had greater blood flow than gluteus maximus (Evans et al., 1975). It is worth noting that these lack of findings may be due to the study design: there were only four subjects in each group, 2 of each sex. Further studies with larger sample size should be conducted in order to confirm the impact of sex on PK of cephradine. We did not identify sex disaggregated PD data in the literature, and the ADR incidence reported in Vigibase fell within the bioequivalence window. Thus it is not possible to define the impact of sex differences on cephradine PK on efficacy, but the effect on safety may not be clinically significant.

Eltanolone is a steroid drug which exerts a hypnotic effect by activating gamma-Aminobutyric Acid-A (GABA_A_) receptors (Brewster et al., 1995; U.S. Department of Health and Human Services, 2004). Eltanolone has never been marketed as an anaesthetic due to undesirable side effects, such as convulsions (Jürgen Schüttler, 2004). However studies exploring its clinical application as antiseizure medication (Frye and Scalise, 2000) motivate the consideration of potential sex differences of PK and PD. As demonstrated by Dale et al., 1999, the eltanolone Vd at steady state is significantly higher in females than males (Dale et al., 1999). The increased distribution of this lipophilic compound in females could be due increased body fat percentage in females (Gandhi et al., 2004; Chu, 2014). An animal PD study conducted by Brewster et al., 1995, reported lower eltanolone efficacy (defined as induction time and sleeping time) and toxicity (defined as LD_50_) in female rats (Brewster et al., 1995). Females have lower expression of the GABA_A_ receptor subunit α1, (Pandya et al., 2019), which may alter its activation threshold and could in turn impact eltanolone efficacy. Preclinical results from Brewser et al., 1995, suggest it is important to investigate sex differences in efficacy and toxicity in humans.

Heparin is an anticoagulant prescribed for treatment of thromboembolism and coronary thrombosis (Campbell et al., 1998). Campbell et al., 1998, reported higher heparin plasma concentration in females than males (Campbell et al., 1998). This could be due to the hydrophilic properties of heparin and the increased body fat percentage in women, resulting in a lower volume of distribution (Gandhi et al., 2004; Fang and Tang, 2020). This has been demonstrated for other water soluble drugs, such as muscle relaxant, which has lower volume of distribution, resulting in higher plasma concentration in females (2). No sex differences in heparin clearance have been reported. Heparin clearance is mediated by the reticuloendothelial system and endothelial cells at low dose, and renal excretion at high dose (51). Future studies may wish to consider reticuloendothelial and renal clearance in both sex groups.

In the same study, females also exhibited higher activated partial thromboplastin time (APTT, an indicator of anticoagulant effect) than males (Campbell et al., 1998). The resulting clotting reduction could explain the previously reported increased incidence of heparin-associated bleeding in females (Landefeld and Beyth, 1993). Despite this report of increased heparin-associated bleeding in females, the overall Vigibase sex ratio (which considers all ADR combined) does not support a significant difference by sex. However, the mechanism underlying the sex differences in the anticoagulant effect of heparin remain unclear. APTT is usually shortened by increased concentration of fibrinogen and coagulation factors, such as FVIII, in the presence of heparin (Mitsuguro et al., 2015). Healthy women have demonstrated higher FVIII and fibrinogen levels than males (Kain et al., 2003). This may result in shorter APTT in females however it contradicts the findings of Campbell et al., 1998. Future studies could include measurement of baseline concentration of coagulation proteins and the plasma protein binding profile of heparin to explore the drivers of sex differences in PK and PD. Studies aiming to understand sex differences in PD and safety may benefit from examining different end points separately (if sex effects are present for some metric but not others).

Linezolid is an oxazolidinone antibiotic used to treat infections by Gram-positive bacteria, such as *Staphylococcus aureus-*induced pneumonia (Hashemian et al., 2018). One of the major side effects associated with the use of linezolid is thrombocytopenia (reduced platelet count) (Sasaki et al., 2011; Kaya Kilic et al., 2019). Linezolid AUC and treatment duration were found to be correlated with reduced platelet count (Kaya Kilic et al., 2019). Recently, a retrospective study conducted by Kaya Kilic et al. (2019), reported that the proportion of patients with thrombocytopenia was greater in males than females (Kaya Kilic et al., 2019), consistent with the increased linezolid-induced ADR reported in Vigibase for males. We therefore hypothesize that linezolid AUC is higher in females than males. However, this is in contrast with the findings of Sisson et al., 2002 (Sisson et al., 2002), who report 60% higher AUC in females. This discrepancy could be due to small sample size in Sisson et al., 2002, (Sisson et al., 2002), leading to findings with low generalizability. Future studies aiming to comprehensively characterise the effect of sex on linezolid PK and PD may wish to explore PK and PD within the same study, and include additional parameters such as AUC, minimum inhibitory concentration or platelet count as a continuous variable rather than categorical thrombocytopenia (Craig, 2003; Tsuji et al., 2017).

## Conclusion

The impact of sex on PK and PD is becoming increasingly recognized as more examples of sex-specific differences in PK and PD are reported. Despite increased enrolment of female participants in recent clinical studies, knowledge gaps of sex differences in PK and PD for many compounds remain, particularly for anticancer drugs. We identified a total of 25 drugs with ≥50% sex differences in PK across oncology and other therapeutic areas. The trend noted in all examples (except for zidovudine, cephradine, cyclosporine and remifentanil) found female subjects exhibit higher exposure than males (characterised by higher AUC, Cmax, lower CL). Higher exposure in females often translated to sex-related differences in PD, efficacy or ADR. The impact of exposure differences on PD, ADR and efficacy were discussed for 9 non-cancer drugs. Among the 9 studied compounds, only zolpidem currently requires a dose adjustment based on sex. We cannot conclude on the requirement of dose adjustment for other compounds due to the limitations of the studies described herein. For example, there is insufficient data to define the clinical impact of observed sex differences in PK and the molecular mechanisms which drive them. These data gaps emphasise the need for further exploration of the impact of sex on PK and PD. Future studies on these compounds with large sample sizes and sex-disaggregated dose-response measurements will enhance our understanding of sex-specific variation in drug safety and efficacy.

In order to focus our analysis on drugs where differences in PK is most likely to have clinical impact, we only included drugs which had a minimum of 50% difference between males and females for at least one PK parameter. This threshold may not be appropriate for drugs with a narrow therapeutic index, where smaller differences may have clinically relevant impacts on toxicity. For example, clozapine plasma levels are correlated with clinical outcomes (Khosravan et al., 2016; Tsuji et al., 2017). Lower clozapine clearance in females could increase ADR risk (Khosravan et al., 2016; Tsuji et al., 2017), hence a lower dose is recommended for females (Tsuji et al., 2017). Hence particular attention to sex differences in PK and implications for sex-specific dose adjustments should be paid to drugs with a narrow therapeutic index.

In addition to *in vivo* studies, a balanced inclusion of cells and animal models of both sex groups will allow identification of sex differences early in the drug discovery pipeline. Furthermore, sex differences in drug metabolism and the PK of active metabolites should also be considered in study design to fully address the sex differences in PK of prodrugs. Robust conclusions from such trials can inform prescribing information to improve patient benefit, reduce ADR and target the right patient population. Ultimately, an improved understanding of the variation in safety and efficacy associated with sex as a biological variable will help guide drug development and prescribing information, accelerating the delivery of the right dose to the right patient.

## Data Availability

The original contributions presented in the study are included in the article/Supplementary Material, further inquiries can be directed to the corresponding author.
