# Supplementary material for: Harnessing Clinical Trial and Real-World Data Towards an Understanding of Sex Effects on Drug Pharmacokinetics, Pharmacodynamics and Efficacy
Source: Front Pharmacol. 2022 Jun 6;13:874606. doi: 10.3389/fphar.2022.874606 (PMC9207260; doi:10.3389/fphar.2022.874606)
Supplement: Supplementary file 1 [file Table1.DOCX]

**Table S1 Summary of studies being used for sex stratified PK data of anticancer drugs**

| Anticancer drugs | Anticancer therapeutic class | Reference |
| --- | --- | --- |
| Tamoxifen | Antineoplastic Hormonal | (1) |
| Sunitinib | Kinase Inhibitors | (2) |
| Rituximab | Antineoplastic Biologics | (3) |
| Panitumumab | Immunomodulators Biologics | (4) |
| Obinutuzumab | Antineoplastic Biologics | (5) |
| Elotuzumab | Antineoplastic Biologics | (6) |
| Durvalumab | Immunomodulators Biologics | (7) |
| Doxorubicin | Anthracyclines | (8) |
| Crizotinib | Kinase Inhibitors | (9) |
| Cabozantinib | Kinase Inhibitors | (10) |
| Bevacizumab | Antineoplastic Biologics | (11) |
| Axitinib | Kinase Inhibitors | (12) |
| 5-fluorouracil | Antimetabolites | (13) |

References

1. Administration FaD. Medical Review and Clinical Pharmacology and Biopharmaceutics Review(S) 2005 [

2. Khosravan R, Motzer RJ, Fumagalli E, Rini BI. Population Pharmacokinetic/Pharmacodynamic Modeling of Sunitinib by Dosing Schedule in Patients with Advanced Renal Cell Carcinoma or Gastrointestinal Stromal Tumor. Clin Pharmacokinet. 2016;55(10):1251-69.

3. Muller C, Murawski N, Wiesen MH, Held G, Poeschel V, Zeynalova S, et al. The role of sex and weight on rituximab clearance and serum elimination half-life in elderly patients with DLBCL. Blood. 2012;119(14):3276-84.

4. Ma P, Yang BB, Wang YM, Peterson M, Narayanan A, Sutjandra L, et al. Population pharmacokinetic analysis of panitumumab in patients with advanced solid tumors. J Clin Pharmacol. 2009;49(10):1142-56.

5. Gibiansky E, Gibiansky L, Carlile DJ, Jamois C, Buchheit V, Frey N. Population Pharmacokinetics of Obinutuzumab (GA101) in Chronic Lymphocytic Leukemia (CLL) and Non-Hodgkin's Lymphoma and Exposure-Response in CLL. CPT Pharmacometrics Syst Pharmacol. 2014;3:e144.

6. Gibiansky L, Passey C, Roy A, Bello A, Gupta M. Model-based pharmacokinetic analysis of elotuzumab in patients with relapsed/refractory multiple myeloma. J Pharmacokinet Pharmacodyn. 2016;43(3):243-57.

7. Ouellet D, Gibiansky E, Leonowens C, O'Hagan A, Haney P, Switzky J, et al. Population pharmacokinetics of dabrafenib, a BRAF inhibitor: effect of dose, time, covariates, and relationship with its metabolites. J Clin Pharmacol. 2014;54(6):696-706.

8. Dobbs NA, Twelves CJ, Gillies H, James CA, Harper PG, Rubens RD. Gender affects doxorubicin pharmacokinetics in patients with normal liver biochemistry. Cancer Chemother Pharmacol. 1995;36(6):473-6.

9. Wang E, Nickens DJ, Bello A, Khosravan R, Amantea M, Wilner KD, et al. Clinical Implications of the Pharmacokinetics of Crizotinib in Populations of Patients with Non-Small Cell Lung Cancer. Clin Cancer Res. 2016;22(23):5722-8.

10. Miles D, Jumbe NL, Lacy S, Nguyen L. Population Pharmacokinetic Model of Cabozantinib in Patients with Medullary Thyroid Carcinoma and Its Application to an Exposure-Response Analysis. Clin Pharmacokinet. 2016;55(1):93-105.

11. Lu JF, Bruno R, Eppler S, Novotny W, Lum B, Gaudreault J. Clinical pharmacokinetics of bevacizumab in patients with solid tumors. Cancer Chemother Pharmacol. 2008;62(5):779-86.

12. Tortorici MA, Cohen EE, Pithavala YK, Garrett M, Ruiz-Garcia A, Kim S, et al. Pharmacokinetics of single-agent axitinib across multiple solid tumor types. Cancer Chemother Pharmacol. 2014;74(6):1279-89.

13. Mueller F, Buchel B, Koberle D, Schurch S, Pfister B, Krahenbuhl S, et al. Gender-specific elimination of continuous-infusional 5-fluorouracil in patients with gastrointestinal malignancies: results from a prospective population pharmacokinetic study. Cancer Chemother Pharmacol. 2013;71(2):361-70.
