# Supplementary material for: Harnessing Clinical Trial and Real-World Data Towards an Understanding of Sex Effects on Drug Pharmacokinetics, Pharmacodynamics and Efficacy
Source: Front Pharmacol. 2022 Jun 6;13:874606. doi: 10.3389/fphar.2022.874606 (PMC9207260; doi:10.3389/fphar.2022.874606)
Supplement: Supplementary file 2 [file Table2.DOCX]

**Table S2 Summary of patient demographics and study designs of drugs of which the 90% CI fell outside the bioequivalence window**

| **Drug (administration route)** | **Number of subjects (female/male)** | **Age** | **Duration (after administration)** | **Single/ multiple dose** | **Studied population** | **References** |
| --- | --- | --- | --- | --- | --- | --- |
| Doxorubicin (intravenous) | 21/6 | 25-75 | 48 hours | Single | Cancer patients | (1) |
| Tamoxifen (oral) | 30/24 | 18-45 | 21 days | Single | Healthy volunteers | (2) |
| Linezolid (oral) | 7/8 | 21-38 | 48 hours | Single | Healthy volunteers | (3) |
| Cephradine (intramuscular injection) | 2/2 | 19-28 | 8 hours | Single | Healthy  volunteers | (4) |
| Saquinavir (oral) | 29/157 | 35-46 | 12 hours | Multiple | Patients with documented HIV infection | (5) |
| Naltrexone (oral) | 73/84 | 34-56 | 1 week | Single | Smokers with 10ppm or more carbon monoxide (CO) level | (6) |
| Ezetimibe (oral) | 6/5 | 21-24 | 96 hours | Single | Healthy volunteers | (7) |
| Zidovudine (oral) | 18/18 | 22-52 | 8 hours | Multiple | HIV seropositive subjects | (8) |
| Zidovudine (intravenous) | 17/17 | 22-52 | 8 hours | Single | HIV seropositive subjects | (8) |
| Tonapofylline (oral) | 8/6 | 18-45 | 96 hours | Single | Healthy volunteers | (9) |
| Labetalol (oral) | 5/14 | 40-63 | 12 hours | Multiple | Hypertensive patients | (10) |
| Cyclosporine (intravenous) | 5/6 | 17-29 | 24 hours | Single | Healthy African American volunteers | (11) |
| Cyclosporine (oral) | 5/6 | 17-29 | 24 hours | Single | Healthy African American volunteers | (11) |
| Ibuprofen (oral) | 10/10 | 18-30 | 8 hours | Single | Heathy volunteers | (12) |
| R-mephobarbital (oral) | 6/6 | 19-25 | 12 hours | Single | Healthy volunteers | (13) |
| Eltanolone (intravenous) | 9/12 | 26-45 | 12 hours | Single | Healthy patients (n=18), patients with mild systemic diseases (n=3) | (14) |
| Tirilazad (intravenous) | 6/6 | 23-42 | 72 hours | Single | Healthy volunteers | (15) |
| Flecainide (oral) | 7/7 | 45-77 | 24 hours | Single | Patients with supraventricular tachyarrhythmia | (16) |
| Human Rho(D) immunoglobulin (intramuscular injection) | 8/10 | 22-44 | 35 days | Single | Healthy D-negative volunteers | (17) |

References

1. Dobbs NA, Twelves CJ, Gillies H, James CA, Harper PG, Rubens RD. Gender affects doxorubicin pharmacokinetics in patients with normal liver biochemistry. Cancer Chemother Pharmacol. 1995;36(6):473-6.

2. Administration FaD. Medical Review and Clinical Pharmacology and Biopharmaceutics Review(S) 2005 [

3. Sisson TL, Jungbluth GL, Hopkins NK. Age and sex effects on the pharmacokinetics of linezolid. Eur J Clin Pharmacol. 2002;57(11):793-7.

4. Vukovich RA, Brannick LJ, Sugerman AA, Neiss ES. Sex differences in the intramuscular absorption and bioavailability of cephradine. Clin Pharmacol Ther. 1975;18(2):215-20.

5. Fletcher CV, Jiang H, Brundage RC, Acosta EP, Haubrich R, Katzenstein D, et al. Sex-based differences in saquinavir pharmacology and virologic response in AIDS Clinical Trials Group Study 359. J Infect Dis. 2004;189(7):1176-84.

6. Epperson CN, Toll B, Wu R, Amin Z, Czarkowski KA, Jatlow P, et al. Exploring the impact of gender and reproductive status on outcomes in a randomized clinical trial of naltrexone augmentation of nicotine patch. Drug Alcohol Depend. 2010;112(1-2):1-8.

7. Bartlett JA, van der Voort Maarschalk K. Understanding the oral mucosal absorption and resulting clinical pharmacokinetics of asenapine. AAPS PharmSciTech. 2012;13(4):1110-5.

8. Aweeka FT, Rosenkranz SL, Segal Y, Coombs RW, Bardeguez A, Thevanayagam L, et al. The impact of sex and contraceptive therapy on the plasma and intracellular pharmacokinetics of zidovudine. AIDS. 2006;20(14):1833-41.

9. Li Z, TenHoor C, Marbury T, Swan S, Ticho B, Rogge M, et al. Clinical pharmacokinetics of tonapofylline: evaluation of dose proportionality, oral bioavailability, and gender and food effects in healthy human subjects. J Clin Pharmacol. 2011;51(7):1004-14.

10. Johnson JA, Akers WS, Herring VL, Wolfe MS, Sullivan JM. Gender differences in labetalol kinetics: importance of determining stereoisomer kinetics for racemic drugs. Pharmacotherapy. 2000;20(6):622-8.

11. Min DI, Lee M, Ku YM, Flanigan M. Gender-dependent racial difference in disposition of cyclosporine among healthy African American and white volunteers. Clin Pharmacol Ther. 2000;68(5):478-86.

12. Walker JS, Carmody JJ. Experimental pain in healthy human subjects: gender differences in nociception and in response to ibuprofen. Anesth Analg. 1998;86(6):1257-62.

13. Hooper WD, Qing MS. The influence of age and gender on the stereoselective metabolism and pharmacokinetics of mephobarbital in humans. Clin Pharmacol Ther. 1990;48(6):633-40.

14. O Dale HH, K Parivar, E Johansson, M Widman. Pharmacokinetics of eltanolone in male and female patients following intravenous bolus injection. Acta Anaesthsiol Scand 1999;43(4).

15. Hulst LK, Fleishaker JC, Peters GR, Harry JD, Wright DM, Ward P. Effect of age and gender on tirilazad pharmacokinetics in humans. Clin Pharmacol Ther. 1994;55(4):378-84.

16. Carrasco-Portugal Mdel C, Flores-Murrieta FJ. Gender differences in the oral pharmacokinetics of fluconazole. Clin Drug Investig. 2007;27(12):851-5.

17. Jilma-Stohlawetz P, Reiter RA, Panzer S, Horvath M, Eppel W, Jilma B. Pharmacokinetics (PK) of S/D treated anti-D immunoglobulin after intramuscular injection in healthy volunteers: gender differences in PK. Transfus Apher Sci. 2005;33(2):135-40.
