# Supplementary material for: Harnessing Clinical Trial and Real-World Data Towards an Understanding of Sex Effects on Drug Pharmacokinetics, Pharmacodynamics and Efficacy
Source: Front Pharmacol. 2022 Jun 6;13:874606. doi: 10.3389/fphar.2022.874606 (PMC9207260; doi:10.3389/fphar.2022.874606)
Supplement: Supplementary file 3 [file Table3.DOCX]

Table S3 Summary of patient demographics and study designs in PK studies of drugs of which the 90% CI fell inside the bioequivalence window

| **Drug (administration route)** | **Number of subjects (female/male)** | **Age** | **Duration (after administration)** | **Single/ multiple dose** | **Studied population** | **References** |
| --- | --- | --- | --- | --- | --- | --- |
| Alosetron | 12/12 | 66-78 | 12 hours | Single | Healthy volunteers | (1) |
| Iptakalim hydrochloride | 6/6 | 18-40 | 30 hours | Single | Healthy volunteers | (2) |
| Remifentanil | 5/10 | 45-69 | 143 hours | Multiple | Patients who received extracorporeal membrane oxygenation (ECMO) support | (3) |
| Torasemide | 7/17 | 48-84 | 24 hours | Single | Patients with arterial hypertension (n=18) or class II or III congestive heart failure (n=6) | (4) |
| Zolpidem | 11/13 | 21-44 | 12 hours | Single | Healthy volunteers | (5) |
| Lidocaine | 9/9 | 19-30 | 8 hours | Single | Healthy volunteers | (6) |
| Methylpredinsolone | 6/6 | 31-49 | 32 hours | Single | Healthy volunteers | (7) |
| Propranolol | 13/15 | 25-29 | 24 hours | Single | Healthy volunteers | (8) |
| Unfractionated heparin | 105/93 | 64-71 | 4-6 hours | Multiple | Patients with proximal deep vein thrombosis | (9) |
| Pravastatin | 8/8 | 21-25 | 12 hours | Single | Healthy volunteers with the homozygous c.521TT genotype | (10) |

References

1. Koch KM, Palmer JL, Noordin N, Tomlinson JJ, Baidoo C. Sex and age differences in the pharmacokinetics of alosetron. Br J Clin Pharmacol. 2002;53(3):238-42.

2. Cai Y, Chai D, Pei F, Fang Y, Wang R, Liang BB, et al. Single-dose pharmacokinetics and safety of iptakalim hydrochloride in Chinese healthy volunteers. J Pharm Pharmacol. 2012;64(3):337-43.

3. Yang S, Noh H, Hahn J, Jin BH, Min KL, Bae SK, et al. Population pharmacokinetics of remifentanil in critically ill patients receiving extracorporeal membrane oxygenation. Sci Rep. 2017;7(1):16276.

4. Werner D, Werner U, Meybaum A, Schmidt B, Umbreen S, Grosch A, et al. Determinants of steady-state torasemide pharmacokinetics: impact of pharmacogenetic factors, gender and angiotensin II receptor blockers. Clin Pharmacokinet. 2008;47(5):323-32.

5. Greenblatt DJ, Harmatz JS, Singh NN, Steinberg F, Roth T, Moline ML, et al. Gender differences in pharmacokinetics and pharmacodynamics of zolpidem following sublingual administration. J Clin Pharmacol. 2014;54(3):282-90.

6. Wing LM, Miners JO, Birkett DJ, Foenander T, Lillywhite K, Wanwimolruk S. Lidocaine disposition--sex differences and effects of cimetidine. Clin Pharmacol Ther. 1984;35(5):695-701.

7. Lew KH, Ludwig EA, Milad MA, Donovan K, Middleton E, Jr., Ferry JJ, et al. Gender-based effects on methylprednisolone pharmacokinetics and pharmacodynamics. Clin Pharmacol Ther. 1993;54(4):402-14.

8. Walle T, Walle UK, Cowart TD, Conradi EC. Pathway-selective sex differences in the metabolic clearance of propranolol in human subjects. Clin Pharmacol Ther. 1989;46(3):257-63.

9. Campbell NR, Hull RD, Brant R, Hogan DB, Pineo GF, Raskob GE. Different effects of heparin in males and females. Clin Invest Med. 1998;21(2):71-8.

10. Mikko Niemi MD MKPM, Pertti J. Neuvonen MD. SLCO1B1 polymorphism and sex affect the pharmacokinetics of pravastatin but not fluvastatin. Clinial Pharmacology and Therapeutics 2006;80(4).
